# 

**Published:** 1896-12-12

**Authors:** 


### NOTICE TO CORRESPONDENTS.

All MS., Letters, Books for Review, and other matters intended for the Editor should be addressed The Editor, "The Hospital," The Hospital Building, 28 & 29, Southampton Street, Strand, W.C.

The Editor cannot undertake to return rejected MS., even when accompanied by stamped directed envelope.

**Notice to Advertisers and Subscribers.**

All Advertisements, Orders for Copies of The Hospital, and Business Communications should be addressed to **THE MANAGER, (Not to The Editor),** The Hospital, The Hospital Building, 28 & 29, Southampton Street, Strand, London, W.C.

The Cost of Subscription, post free, is as follows:—Per week, 2½d.; per quarter, 2s. 8d.; per half-year, 5s. 3d.; per annum, 10s. 6d. Foreign:—Per annum, 15s. 2d.; payable, in all cases, in advance.

**NOTICE.—Vol. XX. of "The Hospital" (April to September, 1896), in handsome cloth binding, is now on sale at the Office of this Journal, price 7s. 6d. Cases for binding the Numbers contained in this Volume 1s. 6d. Index to Vol. XX., 2½d., post free. The Monthly Part for December is now ready, Price 6d., by post 8d.**

### Scraps and Gleanings.

The Children's Hospital at Adelaide has been recognised by the Council of the Adelaide University for the purpose of teaching.

The number of cards issued by the Metropolitan Provident Medical Association during the past four years has been 9,857, 10,021, 9,898, and 10,545 resp ctively.

The threatened famine in India is causing great anxiety, and preparations are being made in advance to alleviate some of the suffering which must be entailed.

Miss G. S. Martin Glasse, of Bournemouth, has bequeathed £200 to the Blandford Cottage Hospital to provide a bed in memory of her late father, Mr. W. B. Glasse, Q.C.

The total cost per day per patient, according to the last report of the Royal Victoria Hospital at Montreal, has been 1·29 dols. Each patient on an average remained in the hospital for 32·5 days.

A deficiency (caused to a great extent by the amount expended on sanitary alterations and improvements) of nearly £50 is shown in the accounts to the 30th June, 1896, of Boston Hospital, Lincolnshire.

It may interest our readers to know that the Ideal Publishing Union has in the press, and will issue in a few days, a pamphlet containing extracts from the temperance writings of the late Sir B. W. Richardson.

The cost per patient at the Lintz Green Convalescent Home, which was established for poor patients of the Sunderland Workhouse Hospital and the Borough Fever Hospital is, according to the report, 9s. 0½d. per head per week.

The percentage of the cost of the hospitals and dispensaries of the Central Provinces of India which has had to be borne by the Government, has decreased from 58·54 per cent. in 1878, to a little over 30 per cent. in 1894 and 1895.

During the year 1895-6, no less than 14,044 specimens from the throats of diphtheria patients admitted to the hospitals of the Metropolitan Asylums Board have been examined by the special staff at the scientific laboratories of the Royal College of Surgeons of England, situated on the Victoria Embankment.

The great growth of the work done by the Melbourne General Hospital is well illustrated by the following figures. In 1848, the year the hospital was established, the number of patients treated amounted to 89 in and 98 out patients; in 1858, to 2,013 in and 4,200 out patients; in 1868, to 2,904 in and 19,727 out-patients; in 1878, to 3896 in and 21,647 out patients; 1888, to 4,216 in and 16,635 out patients; and in 1895-6, to 4,590 in and and 15,969 out patients.

The number of patients treated at the 86 hospitals and dispensaries of the Central Provinces of India has largely increased during the past three years. Thus, in 1893, 8,968 in and 1,085,068 out; in 1894, 10,872 in and 1,191,625 out; and in 1895, 12,224 in and 1,249,366 out patients were treated. That is to say, 11·51 per cent. of the population in 1893, 12·65 in 1894, and 13·28 in 1895 received medical relief. The total expenditure increased from Rs.136,583 in 1893, to Rs.172,378 in 1894, and Rs. 178,656 in 1895.

One thousand one hundred and seven insane persons were under treatment in the five native lunatic asylums of Bengal during 1895, as compared with 1,116 in 1894. The cost of treatment was Rs.91,379, against Rs.95,789 in 1894. In the European Lunatic Asylum at Bhawanipur 54 cases were under treatment in 1895, compared with 64 in 1894, and the expenditure amounted to Rs.22,838, against Rs.23,350. The average yearly expenditure on each inmate in the native asylums was Rs.99 6a. 6p., and in the European asylum Rs.601.

The death rate per 1,000 of the population in the towns of Calcutta and Howrah in 1895 was very largely in excess of previous years for which figures are forthcoming. Thus in 1889 the death rate was 28·7 and 22·7 respectively; in 1890, 29·8 and 20·6; in 1891, 31·5 and 18·3; in 1892, 29·6 and 23·7; in 1893, 29·5 and 21·4; and in 1894, 32·9 and 25·9. In 1895, however, it had grown to 39·6 and 42·8 respectively. In Calcutta this is partly due to the exceptionally large mortality caused by the epidemic of small-pox which visited the town in the early months of 1895.

As illustrating the practical use to which the bicycle may be put, the following account of a bicycle accident, taken from the New York *Medical Record*, is of interest: One of the first to reach the unconscious wheelman was a mounted policeman (on his wheel). Acquainting himself with the serious nature of the case he immediately remounted and sent in an ambulance call from the nearest box. Before the spectators had time to realise that any proper steps were being taken, a surgeon with the red cross of his calling upon his sleeve arrived upon his bicycle and took charge of the case, while the ambulance to which he belonged followed with the lesser speed of horse-power propulsion.

### Books Received.

Edward Arnold.

"A Text Book of Nursing." By C. C. Weeks-Shaw.

E. and S. Livingstone, Edinburgh.

Catechism Series: "Midwifery," Parts I. and II.

MacMillan and Co.

"A System of Gynæcology." Edited by T. C. Allbutt, M.D., F.R.S., and W. S. Playfair, M.D., F.R.C.P.

Bailliere, Tindall, and Cox.

"The Progress of Medical Chemistry." By J. L. W. Thudicum, M.D., F.R.C.P.

"Cycling as a Cause of Heart Disease." By George Herschell, M.D.

"A Vest-Pocket Medical Dictionary." By Albert H. Buck, M.D.

**Periodicals.**—Woman's Signal, Cornhill Magazine, British Review, London, Literary Digest, Leisure Hour, Girls' Own Paper Christmas Number, Boys' Own Paper Christmas Number, Sunday at Home, Sunday Hour, Under the Mistletoe, Pears' Annual.

### The Student of Medicine.

### APPOINTMENTS.

W. B. Bell, M.R.C.S., L.R.C.P., appointed Honorary Physician to Wallesey Cottage Hospital. Dr. D. Buchan, appointed House Physician and Surgeon to the Aberdeen Sick Children's Hospital. Surgeon-Major W. H. Burbe, B.A., M.B., B.Ch., appointed Clinical Assistant to the Chelsea Hospital for Women. F. Cowan, M.R.C.S., L.R.C.P.Lond., appointed an Honorary Surgeon to St. Bartholomew's Hospital, Rochester. Mr. E. Davey, appointed Medical Officer for the St. James's Third District of the Dover Union. E. G. Dutton, M.R.C.S., L.S.A., appointed Medical Officer for the Eastern District of the Plymouth Incorporation. F. W. Grant, M.D.Edin., B.Sc., C M., appointed Visiting Medical Officer to the Dr. Gray's Hospital, Elgin, N.B. J. A. K. Griffiths, M.B.Lond., M.R.C.S., L.R.C.P., appointed Junior House Surgeon to the General Infirmary, Macclesfield. Dr. Irvine, appointed Medical Officer for the Montmorris Dispensary District. J. W. H. Jellett, M.D.Dub.,

**THE STUDENTS OF MEDICINE**—*continued.*

M.B., B.Ch., appointed Medical Officer of Health to the Heavitree Urban District Council. G. A. Jelly, M.R.C S.Eng., L.R.C.P.Lond., L.S.A.Lond., appointed Senior House Surgeon to the General Infirmary, Macclesfield. J. C. Keer, M.R.C.S.-Eng., L.S.A., appointed Medical Officer for the Wickham Market District and the Workhouse of the Plomesgate Union. R. H. Kinsey, M.R.C.S.Eng., L S.A., appointed Consulting Surgeon to the Bedford General Infirmary and Fever Hospital. L. H. Pegler, M.D., M.R.C.S., appointed Surgeon to the Metropolitan Throat, Nose, and Ear Hospital, Fitzroy Square, W. Dr. C. B. Potter, appointed Medical Officer of Health to the Ashbourne Rural District Council. A. W. Riddell, M.R.C.S.-Eng., M.R.C.P.Lond., appointed Honorary Pnysician to the New Brighton Convalescent Home for Women and Children. J. H. Sequeira, M D., M.R.C.P.Lond., F.R.C.S.Eng , appointed Medical Registrar to the London Hospital. J. Stewart, J.P., L.R.C.P., L.R.C.S Edin., appointed Medical Officer for the Parish of Inverary and Glenaray, and under the Local Authority of the Burgh of Inverary, for the North Division of the Parish. G. D. Thomson, L.R.C.P.Lond., M.R.C S., appointed Medical Officer of Health to the Ruskington Urban District Council. R. Trimble, L.R.C P., L R C S Edin., appointed Medical Officer for the First District of West Bromwich Union. Mr. Wall, appointed Medical Officer for the Revelsby District of the Horncastle Union. Dr. W. R. White, appointed Medical Officer for the Benenden District of the Cranbrook Union.

### ROYAL NATIONAL PENSION FUND FOR NURSES.

*President*—**H.R.H. THE PRINCESS OF WALES.**

*Chairman*—WALTER H. BURNS, Esq. *Deputy-Chairman*—HENRY C. BURDETT, Esq.

**PENSIONS. SICKNESS. ACCIDENT.**

### Total Invested Funds, £300,000.

Nurses are invited to join the Fund on account of the substantial and exceptional advantages which it offers them and which *they cannot obtain elsewhere.* The following are the chief points:—

1. **The Fund is Mutual and essentially Co-operative.**

2. **Easy Payment of Premiums.**

   Nurses can pay their premiums monthly or otherwise as best suits their convenience.

3. **The Fund is open to every Nurse.**

   Nurses can assure for Pensions of any amount commencing at any age.

4. **An Investment and Savings Bank.**

   Those entering under the returnable scale can have their premiums returned to them with compound interest, less a small deduction for working expenses. Interest allowed on deposits.

5. **Additions to Pensions.**

   Besides the Pension entered for, substantial additions thereto may be anticipated in the shape of bonuses.

6. **Sickness and Accident Assurance.**

   Policies are issued in connection with Pension policies assuring 10s., 15s., or 20s. a week in cases of incapacity from work through sickness or accident.

Full particulars to be obtained from THE SECRETARY,
ROYAL NATIONAL PENSION FUND FOR NURSES,
28, FINSBURY PAVEMENT, LONDON, E.C.

